# Towards food security: Exploring the spatio-temporal dynamics of soybean in India

**DOI:** 10.1371/journal.pone.0292005

**Published:** 2024-05-09

**Authors:** Meghavi Prashnani, Buddheshwar Dupare, Krishna Prasad Vadrevu, Christopher Justice

**Affiliations:** 1 Department of Geographical Sciences, University of Maryland, College Park, MD, United States of America; 2 Indian Council of Agricultural Research (ICAR), Indian Institute of Soybean Research, Indore, Madhya Pradesh, India; 3 NASA Marshall Space Flight Center, Huntsville, Alabama, United States of America; IISS: Indian Institute of Soil Science, INDIA

## Abstract

India is the world’s largest edible oil importer, and soybean oil accounts for a major portion of those imports, with implications for the Indian economy. Despite being the 4th largest globally in terms of harvested soybean area and 5th largest in terms of production, India is still heavily dependent on imports to meet the vegetable oil requirement for its population. It is therefore imperative to understand the dynamics and trends in India’s soybean production to help the country achieve self-sufficiency in edible oils. This study provides the first spatially explicit analysis of soybean in India, using long-term spatial and temporal statistics at national and subnational levels, using spatial and temporal statistical analysis models to examine the historical trends and its future prospects. Our analysis details the overall soybean expansion across the country and the increase in production but we also note that the annual growth rate has declined in each consecutive decade even though the area continues to expand. The average national yield has been stagnant at around 1 T/Ha but for some of the low-producing districts, a higher yield of more than 3 T/ha is reported. For most major producing districts, soybean yields are below 1.5 T/Ha. The state of Madhya Pradesh which was the major soybean producer is now matched by the state of Maharashtra in terms of production, however, Madhya Pradesh still has the largest area under soybean. We analyzed soybean hotspot expansion in India and found that the mean center of the soybean area and production has shifted approximately 93 km towards the south and 24 km to the west as the crop is rapidly being adopted in the southern and western parts of India expanding the hotspot in these parts. District-level analysis showed that the total number of districts constituting hotspots of soybean cultivation in India has increased from 29 to 42 in three decades. Furthermore, analysis of soybean oil and meal consumption with respect to the national population, import, export, domestic production, GDP per capita, and price of soybean oil and meal suggests that soybean oil and meal are highly correlated with GDP per capita and population, indicating that consumption of soybean oil and meal is likely to increase as GDP per capita increases, and future demand is expected to rise with the anticipated growth in the Indian population. Increased soybean production can play a significant role in increasing national food security for India and reducing dependence on foreign oil imports and also help the economy with soy meal exports. Understanding the spatiotemporal variability in area and yield will help target interventions to increase production. Given the overall low yields with high variability in production, particularly in recent years primarily due to successive extreme rains and droughts in major producing districts and the overall need to increase production to meet the country’s demand, there is a pressing need for government policies and research aimed at narrowing the yield gap and developing soybean varieties that are more productive and resilient to climate change.

## Introduction

The soybean or soya bean (*Glycine max* L. Merrill) is a legume native to East Asia, widely grown for its edible bean, which has numerous uses. It is high in proteins (38–42%) and edible oil (18–22%) containing major essential amino acids [1, 2]. Soybean meal is a substantial source of protein for livestock feed. A major seed legume, it contributes about two-thirds of the world’s protein concentrates for livestock feeding and 25% of the world’s edible oil.

Cultivation of yellow soybean was introduced in India in the early 1970s and since then its cultivation has grown dramatically, placing it at the number one position among the oilseed crops of India. In India, soybean is mainly consumed in two forms, soybean oil, and soybean meal. Soybean oil is one of the most widely used cooking oils in India. It is a popular choice due to its neutral taste, high smoke point, and health benefits. Soybean oil is also used in the food industry for making margarine, mayonnaise, and salad dressings, as well as in the production of non-food items such as biodiesel, paints, and plastics [3].

Soybean meal is the byproduct of soybean oil extraction and it is gaining domestic importance in India due to its protein richness both for humans and livestock [4, 5]. Soybean meal is primarily used as a protein source in animal feed especially for poultry and swine due to its high amino acid content, digestibility and affordability [6]. Soy meal is gaining popularity in India as a source of protein for humans as well, especially among vegetarians and vegans. Soy meal is a high-quality plant-based protein that contains all the essential amino acids required by the human body [7]. It is also a good source of dietary fiber, vitamins, and minerals. In recent years, there has been an increasing trend towards plant-based diets and a growing awareness of the health and environmental benefits of consuming plant-based protein sources. Indian soymeal is in international demand due to its non-GMO property. Recently, the United States became a major importer of Indian soy products, including soy meal, oilcakes, and organic soybeans, highlighting the diverse and growing demand within the U.S. market [8, 9].

From a farming perspective, soybean is an important crop, as it enhances soil health and fertility by fixing nitrogen in the soil, benefiting successive crops. Some studies also suggest that soybean has the capacity to improve the nutritional situation, enhance the productivity of other crops and protect the environment from the adverse effects of agricultural chemicals [10, 11].

There is a nearly five-decade history of soybean growth and commercialization in India. Soybean was established as a major monsoon season crop (*kharif*) in the rainfed agricultural systems of central and peninsular India. It is sown mostly during the *kharif* (monsoon) season with the onset of the monsoon in June/July and harvested in September/October. It was introduced into India in 1970 and gained popularity mainly in the Malwa region of central India, considered to be the epicenter of soybean cultivation in India [12]. Soybean in India is primarily grown as a rainfed crop on vertisols and associated soils, with an average crop season rainfall of 900 mm, which varies greatly across locations and years [13].

Soybean introduction has led to a transformation in the cropping system, planted in crop rotation with winter (*Rabi*) crops like wheat and chickpea. This has resulted in an enhancement in the cropping intensity and a resultant increase in the profitability per unit land area, leading to the improvement of socioeconomic conditions for the small and marginal farmers [13, 14].

Despite all these positive aspects of soybean production, there is a serious concern regarding soybean yield in India, which currently stands at around 1T/Ha, which is considerably less than other major soybean producers in the world. Being a rainfed crop, soybean in India is highly dependent on monsoon rains and hence is vulnerable to climate change [15]. It is therefore very important to make it climate resilient and to increase its productivity under different types of stress such as drought, flood, hailstorm, and pests [16].

India is heavily dependent on imports to meet its domestic edible oil demand [17, 18], importing nearly 70% of its edible oil from countries such as Indonesia, Malaysia, Russia, and Argentina. Soybean provides the majority of its domestic edible oil production and in the past few decades has proven to be a reliable source of oilseed for India. It is therefore important to study and understand Indian soybean with respect to international production and demand and to help identify global trends in soybean production and consumption, which can be useful for predicting future demand and supply dynamics. This information can help Indian producers and policymakers make informed decisions about production levels, pricing, and trade. Furthermore, an international comparison can provide a benchmark for evaluating the effectiveness of government policies and programs aimed at promoting soybean production. Therefore, this study tries to examine the Indian soybean in an international context to gain insights into the competitiveness, effectiveness, and future prospects of Indian soybean production in the global market.

To date, while there have been a few studies that have explored the temporal aspects of soybean over a short duration or for a limited study area [18], there has not been a comprehensive examination of Indian soybean that considers both its spatial and temporal dimensions at both the national and subnational levels. This study aims to fill that gap by investigating the long-term dynamics of Indian soybean, including its potential drivers and future prospects, through an analysis of its trends over time and space. Specifically, this paper examines:

- National-level soybean trends.
- Soybean dynamics across states, districts and agroclimatic zones.
- Hotspots and cold-spots of soybean production.
- Yield distribution and stagnation.
- Potential drivers of soybean trends and future projections.
- Trends in Indian soybean production with respect to international trends.

The rationale for this study is to examine the dynamics, trends, and future prospects of soybean production in India at both national and subnational scales. The study aims to provide a comprehensive understanding of the spatial and temporal dimensions of Indian soybean, identify potential drivers of soybean trends, and explore the competitiveness and future prospects of Indian soybean in the global market. Additionally, the study aims to contribute to the goal of achieving self-sufficiency in edible oils in India and inform decision-making processes related to soybean production, pricing, and trade. However, obtaining data at the lower administrative level presented challenges due to inconsistencies in format, given that different state departments maintain their own datasets, making data homogenization a complex task. In this study, we utilized district-level, state-level, and national-level crop statistics for all available years with the most reliable data. Efforts were made to address anomalies in the dataset. It is important to note that sub-district level crop statistics at the national level are currently not available in a standardized format, which further highlights the necessity for in-depth research in this area. For subsequent investigations, we intend to conduct a comprehensive study at the district and village levels, using crop statistics complemented by long-term climate and socio-economic data employing spatial econometrics models and advanced Geographic Information System (GIS) tools to gain deeper insights into this subject. Advanced GIS tools will help to analyze, visualize, and interpret spatial and temporal patterns of soybean and their associated factors. However, with the current scenario of limited data availability, this present paper forms the foundation for future research endeavors in the domain of soybean production dynamics in India.

## Data and methods

Two data sources were used for national, state, and district levels analysis, viz. the Directorate of Economics and Statistics (DES), Ministry of Agriculture and Farmers’ Welfare, Government of India and the United States Department of Agriculture (USDA). For national analysis USDA and DES data were used and for state and district analysis DES data was used. The national and state level data were available from 1960, while the district data were only available from 1987 onwards. Data for GDP per capita and population was taken from the World Bank, while data for oil and meal prices were taken from Macrotrends and domestic oil and meal production, consumption, import and export statistics were taken from USDA. To mitigate potential issues with missing data and weather-related anomalies, this study utilized three-year averages around the decade ending years i.e. 1970, 1980, 1990, 2000, 2010, and 2020 for the decadal analysis, for example for 2000 we took the average of 1999 to 2001 to represent that period in time, referred to as circa 2000 or c. 2000, similarly c. 1980 represents the average of the year 1979, 1980 and 1981. During the reporting period, district boundaries changed due to official bifurcation and merger of districts, hence care was taken to harmonize these changes so that a consistent spatial analysis could be conducted.

### National trend analysis using the Mann-Kendall test

The Mann-Kendall test was used to analyze the trend in the national data. It is a non-parametric statistical test used to detect trends in time series data. It tests the null hypothesis that there is no trend in the data against the alternative hypothesis that there is a monotonic trend (either increasing or decreasing). The test statistic is based on the differences between pairs of observations in the time series. If there is a trend, then the signs of the differences will tend to be either all positive or all negative. The Mann-Kendall test statistic is the sum of the signs of the differences, and its distribution under the null hypothesis is known. A large positive or negative value of the test statistic indicates a strong trend in the positive or negative direction, respectively. The p-value of the Mann-Kendall test indicates the strength of evidence against the null hypothesis. A small p-value (e.g., less than 0.05) suggests strong evidence against the null hypothesis and in favor of the alternative hypothesis of a monotonic trend.

The test statistic for the Mann-Kendall test can be calculated using the following formula:

$$S=\sum \left(sgn(x_{i}-x_{j})\right)$$ (1)

Where x_i_ is the i^th^ observation, x_j_ is the j^th^ observation, sgn() is the sign function, which returns -1 if (x_i_-x_j_) is negative, 0 if (x_i_-x_j_) is zero, and 1 if (x_i_-x_j_) is positive. The test statistic S follows a normal distribution with mean 0 and variance:

$$Var(S)=\frac{\left[n*(n-1)(2n+5)-\sum t_{j}(t_{j}-1)*(2t_{j}+5)\right]}{18}$$ (2)

Where n is the number of observations, t_j_ is the number of tied groups in the j^th^ group. The standardized test statistic Z can be calculated as:

$$Z=\frac{\left(S-1\right)}{\surd Var(S)}$$ (3)

Where 1 is the expected value of S under the null hypothesis of no trend. The p-value can be calculated from the standard normal distribution, using the Z statistic and the appropriate tail(s) of the distribution.

### Decadal analysis using CAGR and CV

For the national and state level trend analysis, we divided the data into 5 decades D1 (1970–1980), D2 (1980–1990), D3 (1990–2000), D4(2000–2010), D5(2010–2020) and calculated the Compound Annual Growth Rate (CAGR) and Coefficient of Variation (CV). CAGR is the annualized average rate of growth between two given years, assuming growth takes place at an exponentially compounded rate. The formula for CAGR is given below:

$$CAGR={\left(\frac{v_{final}}{v_{initial}}\right)}^{\frac{1}{t}}-1,$$ (4)

where v_final_ is the last year of the decade, v_initial_ is the beginning year of the decade, t is the number of years which is 10 for decadal analysis.

CV is the measure of the relative variability or dispersion of a dataset. It is calculated by dividing the standard deviation of the dataset by the mean. A low CV indicates that the data points are relatively close to the mean, while a high CV indicates that the data points are more spread out around the mean. The formula for CV is:

CV=(standarddeviation/mean)x100% (5)

### Spatial autocorrelation using Global Moran’s I

Spatial autocorrelation has been widely used to explore the spatial pattern of variables for various applications. It measures how one object is similar to others surrounding it. There are various indices used to measure spatial autocorrelation at different levels, depending on the application. Spatial autocorrelation analysis can identify spatial clusters (positive autocorrelation) and spatial outliers (negative autocorrelation) of a regionalized variable [19]. Global Moran’s I is a measure of the overall clustering of the spatial data. Values of I usually range from −1 to +1 with values tending towards +1 indicating positive spatial auto-correlation and *vice versa*. The z-score measures the degree to which the observed Moran’s I coefficient differs from what would be expected under the null hypothesis of no spatial autocorrelation. A high absolute value of the z-score indicates that the observed Moran’s I coefficient is significantly different from the expected value, suggesting the presence of spatial autocorrelation.

$$Global\;Moran^{\prime }s\;I=\frac{1}{\sum_{i=1}^{n}\sum_{j=1}^{n}w_{ij}}*\frac{\sum_{i=1}^{n}\sum_{j=1}^{n}w_{ij}(x_{i}-\bar{x})(x_{j}-\bar{x})}{\sum_{i=1}^{n}{\left(x_{i}-\bar{x}\right)}^{2}/n}$$ (6)

where I is the Moran’s I statistic, x_i_ is the value of variable at location i, x_j_ is the value of a variable at location j, and w_ij_ is the weight that determines the relationship between i and j.

### Hot spot analysis (Getis-Ord Gi*)

Hotspot analysis was used to identify hotspots of soybean production and how they have shifted over the study duration. The Getis-Ord Gi* statistic can be used to perform hotspot and cold-spot analysis to identify statistically significant clusters of high or low values in a dataset. It is based on the concept of spatial autocorrelation, which refers to the tendency of nearby values to be more similar than those that are further apart. The test measures the degree of spatial association between each feature in a dataset and its neighboring features, and then assigns a score to each feature based on its level of association. G* is high where the sum of values within a neighborhood of a given distance or arrangement is high relative to the global average and negative where the sum of values within a neighborhood are small relative to the global average and approaches 0 at intermediate values [20, 21]. The neighborhood function determines which features are considered neighbors of a specific feature based on their spatial proximity or other defined criteria. The function assigns weights to these neighboring features to measure the degree of association or interaction between them. There are different types of neighborhood functions, depending on the application and analytical requirements: distance-based neighborhood, contiguity-based neighborhood, and kernel-based neighborhood. The appropriate conceptualization of spatial relationships and the best distance band or threshold distance value depend on the specific context and the nature of the data being analyzed [22].

## Results

### Trend analysis at national level

**Fig 1:**
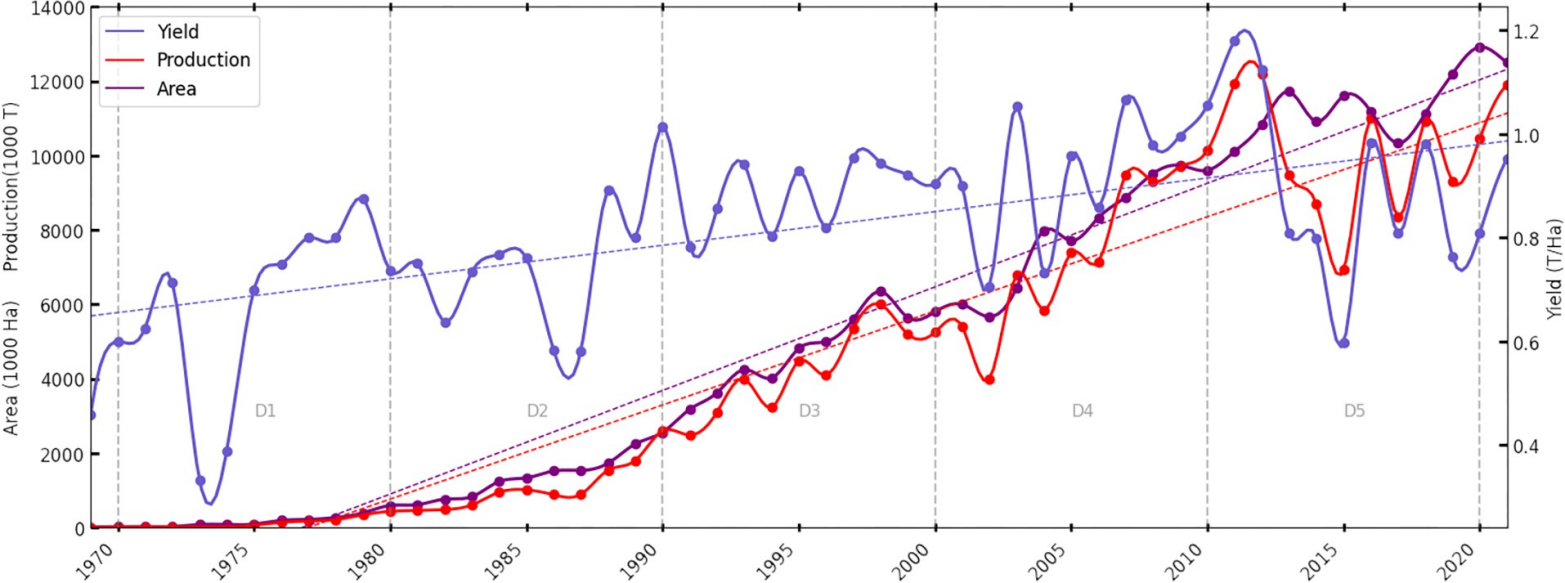
Growth trajectory of soybean at the national level. Area (Hectares), Production (Tonnes) and Yield (Tonnes/Hectare) at the national level for India from 1970 to 2022.

Soybean area, production and yield are plotted annually at the national scale for India from the 1970 to 2022 (Fig 1) which depicts an overall upward trend in area, production and yield, however, the yield and production can be seen to plateau in recent years. To further analyze the trends, we performed the Mann-Kendall test and the results suggest that there is a significant upward trend in soybean area, production, and yield over time. For Area, the Mann-Kendall test yielded a z-score of 10.32 and a very low p-value of 2.2e-16, indicating a strong and statistically significant upward trend in the soybean area over time. The large value of S (1.38e+03) and tau (9.67e-01) further supports the presence of a significant positive trend. Similarly, for production, the Mann-Kendall test yielded a z-score of 9.53 and a very low p-value of 2.2e-16, indicating a strong and statistically significant upward trend in soybean production over time. The large value of S (1.27e+03) and tau (8.93e-01) supports the presence of a significant positive trend. For Yield, the Mann-Kendall test yielded a z-score of 4.81 and a p-value of 1.494e-06, indicating a strong and statistically significant upward trend in soybean yield over time, although it appears to be stagnant over the last decade. The sample estimates show a value of S (6.46e+02) and tau (4.51e-01), indicating a less pronounced but still positive trend.

CAGR and CV in Table 1 and Fig 1 show that the soy harvested area has consistently increased over time, while production has had some significant dips with the highest production achieved in 2012. Average yield increased steadily but flattened out starting in 2012. In the first decade, 1970–80 the total national soy area increased at a CAGR of 34% (from 0.029 MHa to 0.54MHa), while production increased from a CAGR of 38.6% (0.016 MT to 0.42 MT). This was the period of greatest growth in soybean. For each of the subsequent decades the rate of growth in area and production approximately halved. In the last decade (D5), production went into decline (negative growth). The rate of growth in yield decreased steadily with each decade (D1 3.4% to D4 1.7%). During 2010 to 2020, the national growth rate in yield went negative (D5-2.4%) bringing down overall production despite of increase in area under the crop.

**Table 1:** Comparison of soybean area, production and yield using the CAGR (annualized average rate of growth rate between two given years) and CV (standard deviation over mean during each decade) for 5 decades 1970 to 2020. The table has been color-coded with dark green being the highest CAGR of 34% and yellow as lower with a CAGR towards zero or negative CAGR.

CAGR (CV)	D1 (1970–80)	D2 (1980–90)	D3 (1990–2000)	D4 (2000–10)	D5 (2010–20)
Area	33.99% (95%)	17.30% (47%)	8.11% (26%)	5.37% (20%)	2.48% (9%)
Production	38.65% (100%)	18.54% (63%)	8.67% (29%)	7.20% (29%)	-0.04% (16%)
Yield	3.44% (26%)	0.96% (17%)	0.49% (8%)	1.72% (13%)	-2.45% (20%)

Overall, the area, production, and yield have improved in the past five decades, however, the rate of improvement has slowed in each of the five decades consecutively. For the last decade, the yield and production have shown negative CAGR, while the area increased only by 2% CAGR because of the poor yield during last few years at the national level. There is considerable variation in each decade especially for the first decade as shown by the Coefficient of Variance (CV) statistics. In the first decade (1970–80), total of 1786% of increase in area (CAGR 34%) with CV of 95% and total 2469% of increase in production (CAGR 39%) with CV of 100% was reported (S1–S3 Tables in S2 File). For subsequent decades, the CV for the national area was 9%, and 16% for production, which means the area was increasing slowly but production was not stable in the last decade. For the same period, there was negative growth rate in yield (-2.45%) with a CV of 20%, showing the turbulence in yield in recent decades.

### Interstate comparison of soybean

**Fig 2:**
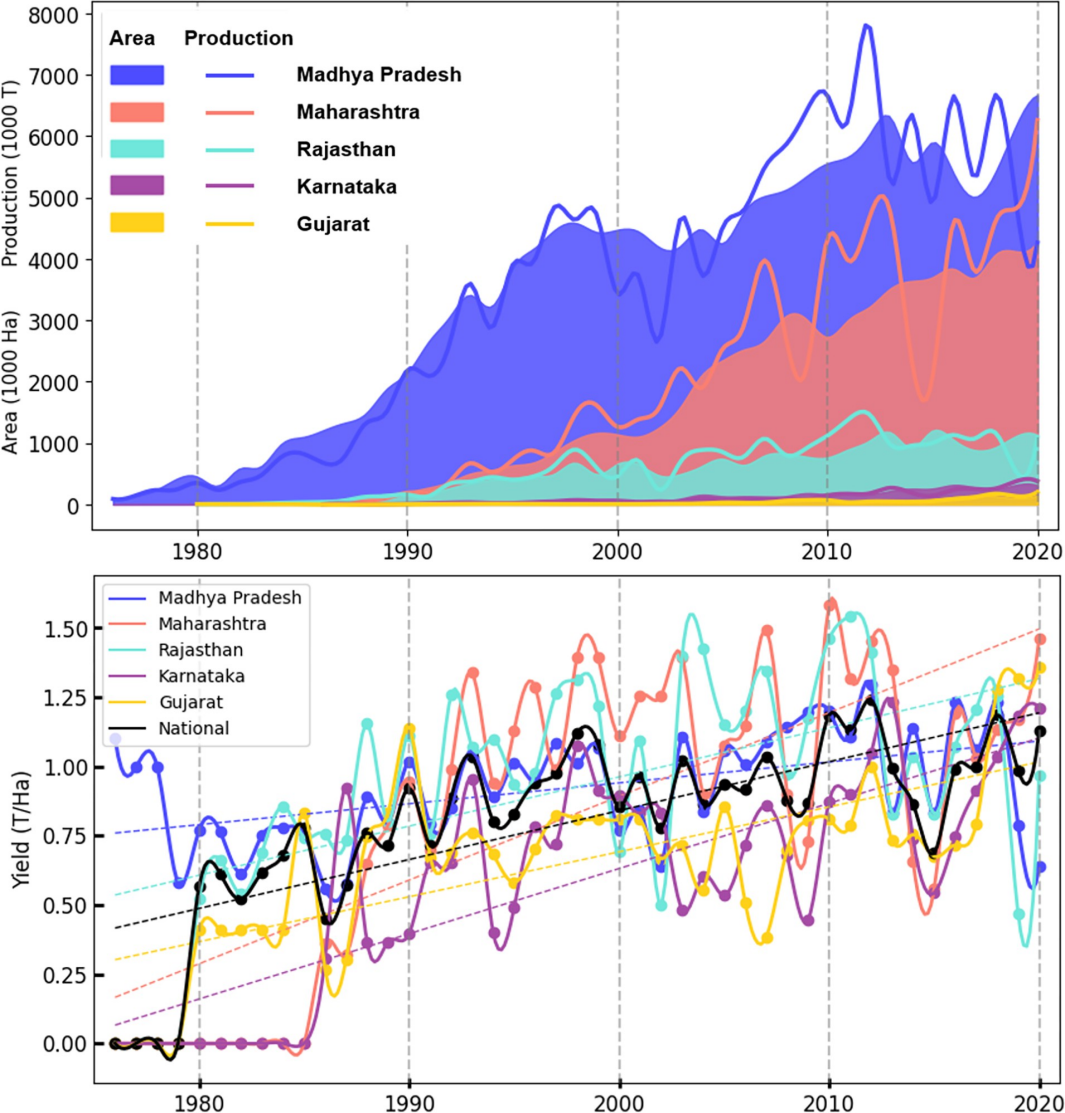
Comparison of soybean area, production and yield at state level for major producing states. (Madhya Pradesh, Maharashtra, Rajasthan, Karnataka, and Gujarat.) In addition, the yield graph shows the average national yield compared to the state yield.

Data were plotted data for the five primary soy-producing states (Madhya Pradesh, Maharashtra, Rajasthan, Karnataka, and Gujarat), which together currently account for more than 98% of national soybean production (Fig 2).

Though soybean was grown traditionally in India as black soybean [12], the yellow soybean was introduced in Madhya Pradesh in the 1970s. The cultivation of soy was subsequently adopted by farmers of Gujarat and Rajasthan around 1980, and Maharashtra around 1990. Although being adopted in Rajasthan and Gujarat earlier than in Maharashtra, the area under soy increased more rapidly in Maharashtra. The rate of growth in Maharashtra began to accelerate around 2000. Madhya Pradesh contributed around 97% of India’s soybean production around 1980 but in 2020, its contribution declined to 52% in terms of sown area and 44% in production. Over the same period, Maharashtra’s contribution increased from 1% to 45% in 2020. Currently Rajasthan contributes 8% and Karnataka 3% of production respectively. Clearly, the crop has drastically expanded in other parts of the country in past two decades and Madhya Pradesh can no longer be called the soybean bowl of India.

**Fig 3:**
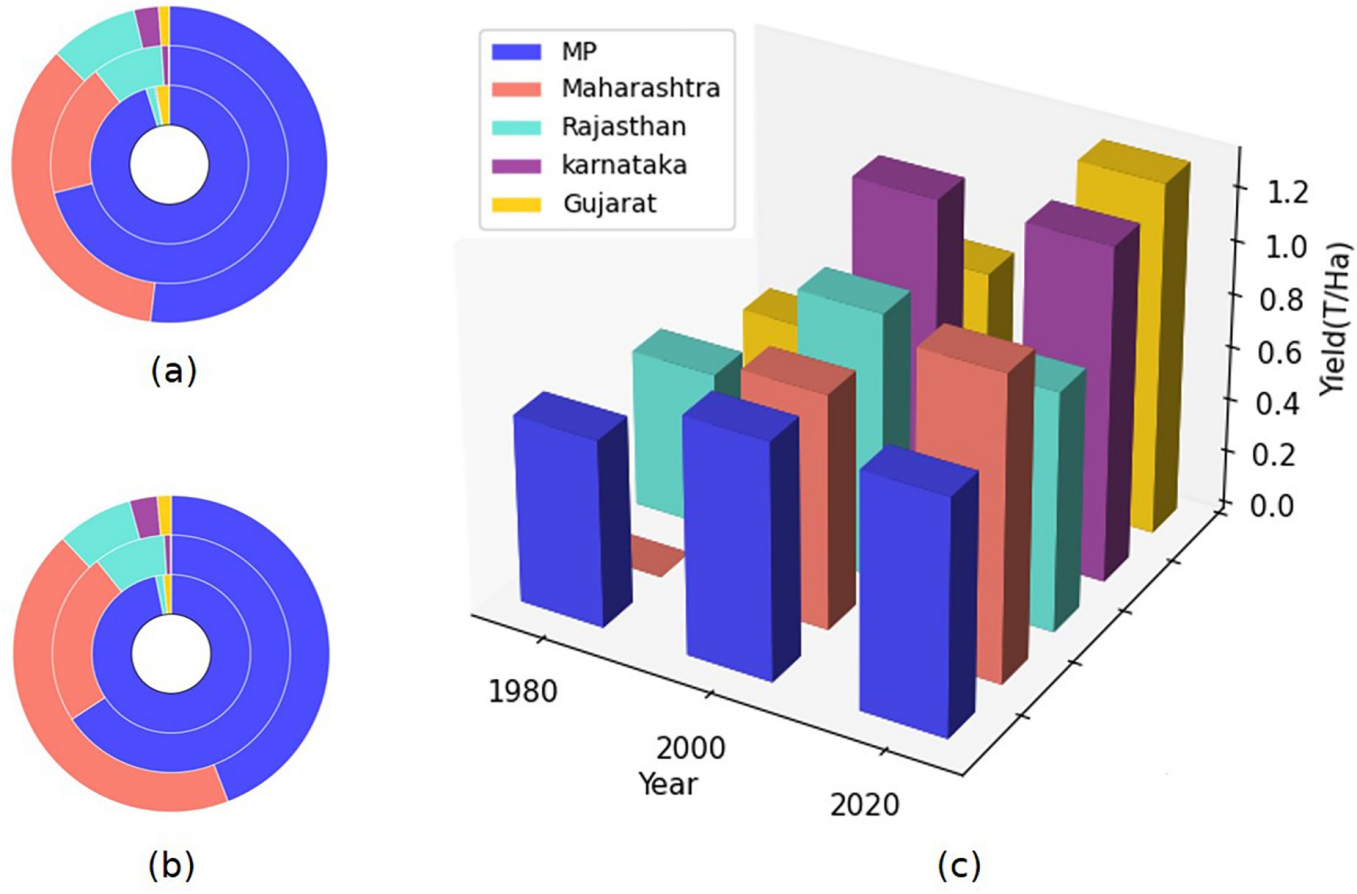
Soybean area, production and yield decadal comparison at the state level. (a) Contribution to national soybean acreage by major states in c1980 inner circle, c2000 middle circle, c2020 outer circle. (b) National soybean production contribution by each state; c1980 inner circle, c2000 middle circle, c2020 outer circle. (c) soybean yield for years c.1980, c2000, c.2020.

Fig 3 shows the decadal analysis of area, production, and yield for 5 major producing states of India. Madhya Pradesh alone was the major producer of soybean contributing more than 95% to the national production but recently Maharashtra is contributing more, despite Madhya Pradesh having a larger area under the crop. This is because of the poor yield in Madhya Pradesh in recent years due to various factors including extreme weather conditions. The overall negative growth of production for the last decade could be attributed to the poor yield due to major flooding and waterlogging during the maturity stage of soybean in 2019 and extensive drought in 2020 in Madhya Pradesh. As a result, we see that although yield declined in Madhya Pradesh, in Maharashtra the productivity continued at 1.3 T/Ha in c.2020. Madhya Pradesh and Rajasthan have had consistent yields over the years averaging around 0.9 T/Ha and 0.7 T/Ha respectively, with some fluctuations but no major increase or decrease. Gujarat has shown a significant increase in yield from c.1980 (0.4T/ha) to c.2020 (1.3 T/Ha).

### Spatial autocorrelation and trend analysis at the district level

**Fig 4:**
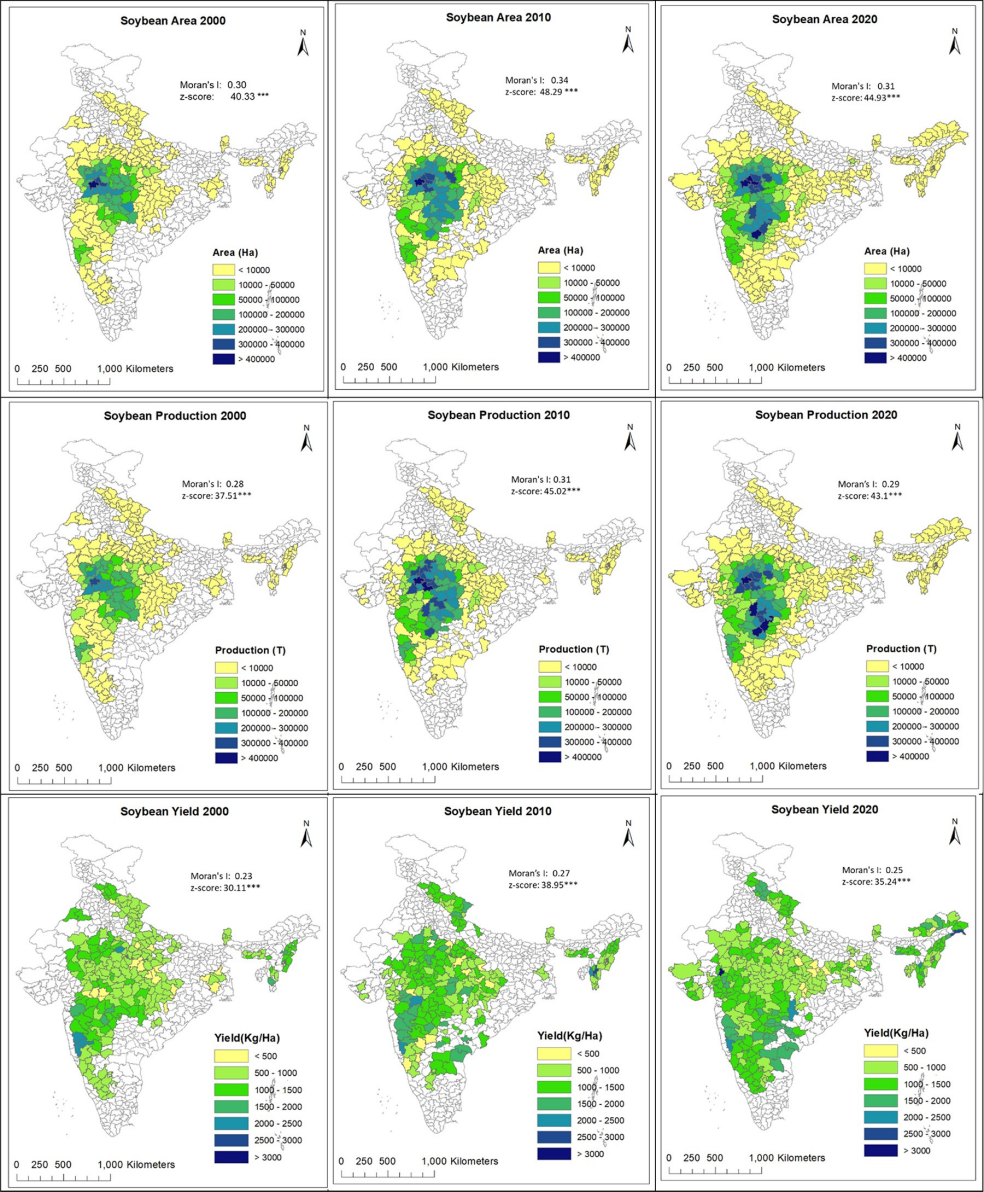
Soybean area, production and yield distribution in India for c. 2000, c. 2010 and c. 2020 along with the Moran’s I value and corresponding z scores. *** represents p value < 0.001.

Fig 4 shows the spatial distribution of area, production, and yield of soybean for three decades along with the Global Moran’s I values and z scores significance level (*** indicates p-value < 0.001).

All three variables (Area, Yield, and Production) show positive spatial autocorrelation in all three years, as indicated by the positive values of Moran’s I coefficient. The z-scores are also very high for all variables and years, with p value less than 0.001 indicating that the observed Moran’s I values are significantly different from what would be expected under the null hypothesis of no spatial autocorrelation. Overall, this suggests that there is a spatial pattern in the distribution of the variables, and nearby observations tend to have similar values.

#### Circa 2000

District-wise distribution and trends show that in c.2000 soybean was concentrated in the Malwa Plateau of Madhya Pradesh State, with the Ujjain District contributing to 0.42 MHa (%) area and 0.36 MT (%) production, followed by Shajapur with 0.31MHa (%) area and 0.28 MT (%) production. In 2000 and 2001 the productivity was very low in the Malwa region and as a result the average productivity is low. Looking at the overall yield c. 2000, the highest yield was observed for Sangli (Maharashtra), Gwalior (Madhya Pradesh) and Satara (Maharashtra) with 2.1 T/Ha, followed by Kolhapur (Maharashtra), Lunglai (Mizoram) with 2.0 T/Ha.

#### Circa 2010

The Malwa region continued to be the leading producer of soybean in India, but by this time, soybean had spread to the adjacent agro-climatic zones including the Vindhyan Plateau and central Narmada Valley in Madhya Pradesh. It had also expanded to the Vidarbha region of Maharashtra and the humid southeastern plains of Rajasthan (Fig 4). The top producing district remained Ujjain (Madhya Pradesh) with an area of 0.45 MHa and production of 0.63 MT, followed by Shajapur, Dewas and Sagar Districts from same agroclimatic zone as Malwa. In Maharashtra, the Amaravati District led with 0.29 MHa area and 0.36 MT production, followed by Nagpur and Yavatmal which all belong to same Vidarbha agro-climatic region. In Rajasthan in 2010, Jhalawar was the leading district, with an area of 0.24 MHa and production of 0.33 MT. In 2010, the highest yields were observed for Aizawl (Mizoram) with 2.5 T/Ha followed by Kolhapur with 2.4 T/Ha, Mamit with 2.23 T/Ha and Jalgaon with 2.1 T/Ha. For same period, the lowest yield was observed for Gulbarga (0.28 T/ha) and Barwani (0.31 T/Ha) followed by Anuppur, Bhind and Chhatarpur all three with 0.41 T/ha. One interesting thing to note here is that most of the poorest yields were observed in districts of Madhya Pradesh, which was the state with the highest overall production during c. 2010.

#### Circa 2020

In the previous three decades, Ujjain and Shajapur districts had been the primary producers, but by 2020 the situation had changed, with Buldana District in Maharashtra State becoming the highest producer with 0.47 MT production, followed by Latur with 0.46MT. In 2020, Ujjain still holds the largest area with 0.48 MHa and production of 0.45 MT, followed by Shajapur with an area of 0.42 MHa and production of 0.37 MT. This is because of the lower yield of 0.93 T/ha for Ujjain and Shajapur, while for Buldana and Latur it was 1.12 T/ Ha. The maximum yield for c.2020 was observed for the Garo Hills Districts of the northeastern states, while the lowest yield was reported for districts of Madhya Pradesh (Singrauli, Satna) and Chhattisgarh (Bemetra, Kabirdham) as shown in Fig 4.

### Yield distribution for 20 major producing districts

**Fig 5:**
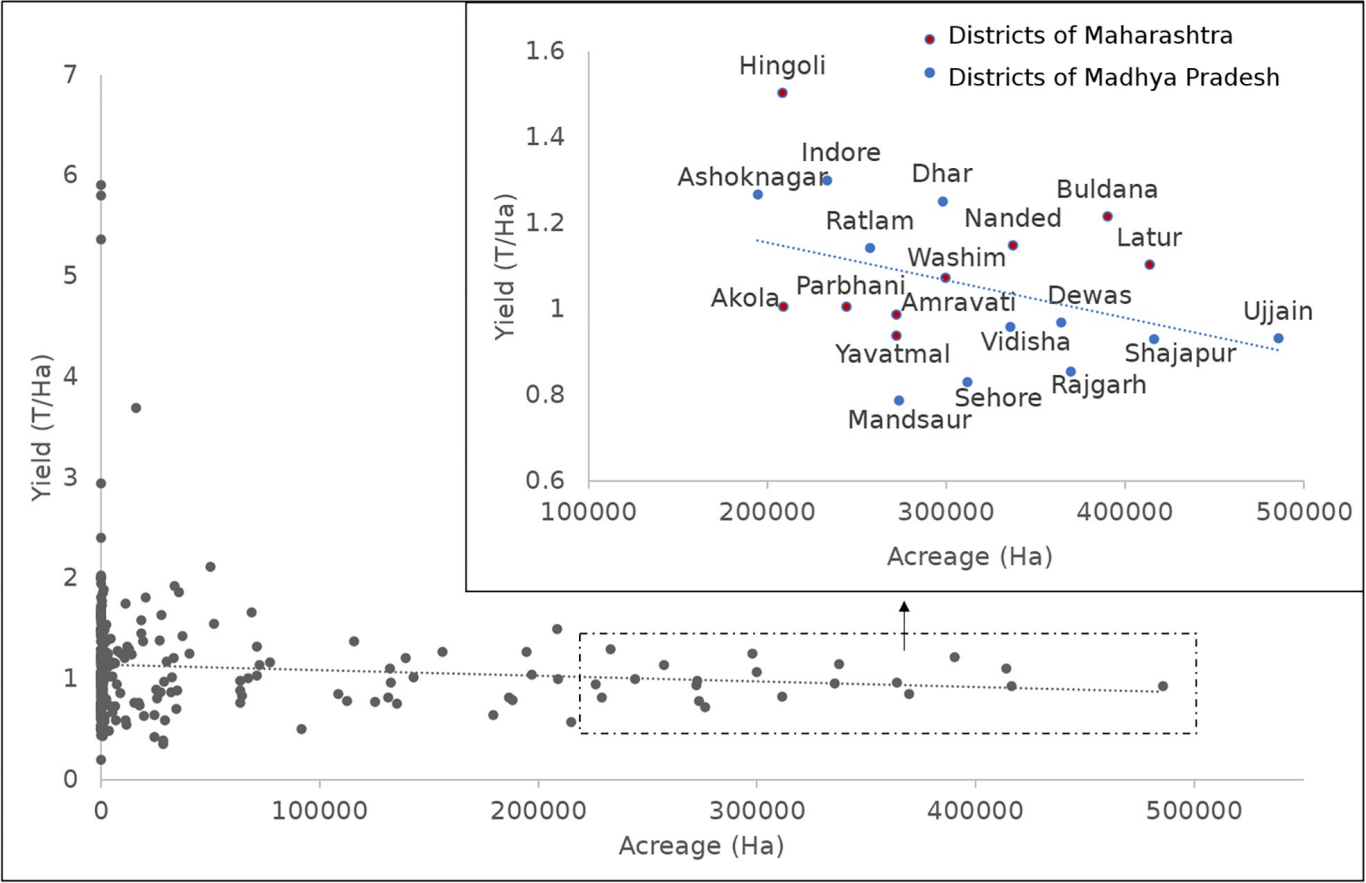
Scatter plot of area vs yield for all districts for c2020 and the zoomed-in window shows the scatter plot for the 20 major producing districts.

To further analyze yield distribution, we compared the area and yield using a scatter plot for all districts and further analyzed the major 20 soybean producing districts in detail. Fig 5 shows a scatter plot between yield and area for all districts cultivating soybean for c.2020. We collected data from all the districts in India that cultivated soybean in the recent years i.e. circa 2020. Using these data, we created scatter plots to visualize the relationship between yield and acreage for each district. Furthermore, from this dataset, we identified the top 20 soybean producing districts for c. 2020 and separately plotted their data in a zoomed version within the same Fig 5. This allowed us to gain more detailed insights into the behavior of the major producers concerning their yield and overall acreage and production. Those 20 districts contribute around 57% of total production in year c 2020. The scatter plot of yield vs. acreage for c.2020 shows that the districts with higher area have lower average yields and the districts reporting highest yield mostly in the northeastern hill region have the lowest area under the soybean crop. Lower yield averages for districts with large areas under soybean crop may be due to the various biotic (pests and diseases) and abiotic factors causing yield losses resulting in a low average. Also, it is noteworthy that there were unusually heavy rainfall in 2019, while in 2000 there was drought in some part of Madhya Pradesh resulting in average lower yield and production for c. 2020 [23]. The impact of weather variability on yield is beyond the scope of this paper and is a topic for future research.

For c. 2020, the highest yield was observed in the northeastern districts of India, while these are not major producers as the area under soybean is considerably lower as compared to central India. The Southwest Garo Hills and West Garo Hills Districts of Meghalaya show the highest yields with 5.9 T/ha and 5.8 T/ha respectively, although the area contribution from these districts is very low. This comparatively high yield with small area could be in part because of the high variability in yield in major producing districts including farms with poor yields in many fields, which would average out to a lower yield for the entire district. Another reason for these yield gaps could be the varieties used or the climatic or soil conditions. The most likely explanation could be that the districts having comparatively less area started cultivation very recently on virgin soils. Further, less area facilitates the farmers to adopt the recommended agronomic practices at the right time and the crop can be managed for insect-pests and diseases. Similarly, the soybean areas of northeastern states have more soil organic carbon content and are thus able to give higher yields, as compared to the high intensity, large area soybean growing states/districts which are comparatively more prone to biotic and abiotic factors. In either case, such yield gaps are an important topic for further research.

Some exceptions can be found, where districts have both good yield and high cultivation area, for example the Satara District in Maharashtra has an area of 0.07 MHa with a yield of 1.7 T/ha, with a production of 0.11 MT, making it one of the major producer districts, despite having less area than some other districts. It is interesting to note that there is no single district from Madhya Pradesh in the top 70 high yield soybean districts. The maximum yield in Madhya Pradesh is observed in Indore with an area of 0.2 MHa and a yield of 1.3 T/Ha. The Barwani District (MP) had the poorest yield of 0.3 T/ha for c. 2000 and 2010, but in 2020 improved to 1.2 T/Ha.

A zoomed-in plot in Fig 5 shows the scatter plot for 20 major producing districts for c.2020. These 20 districts include 9 districts from Maharashtra state and 11 districts for Madhya Pradesh, contributing to 57% of national soybean production in c. 2020. The blue represents the districts in Madhya Pradesh state and red represent the districts in Maharashtra state. The lowest soybean yield is observed in Ujjain (Madhya Pradesh) 0.9 T/Ha while it has the highest area among all districts of India. The highest yield was observed for Hingoli District (Maharashtra) 1.5 T/ ha with area 0.2 MHa. For most of the top producing districts the yield is around 1.1 T/ha. For Madhya Pradesh State, Indore and Dhar districts reported the highest yield.

### Hotspot analysis with respect to agroclimatic zones and spatial mean

**Fig 6:**
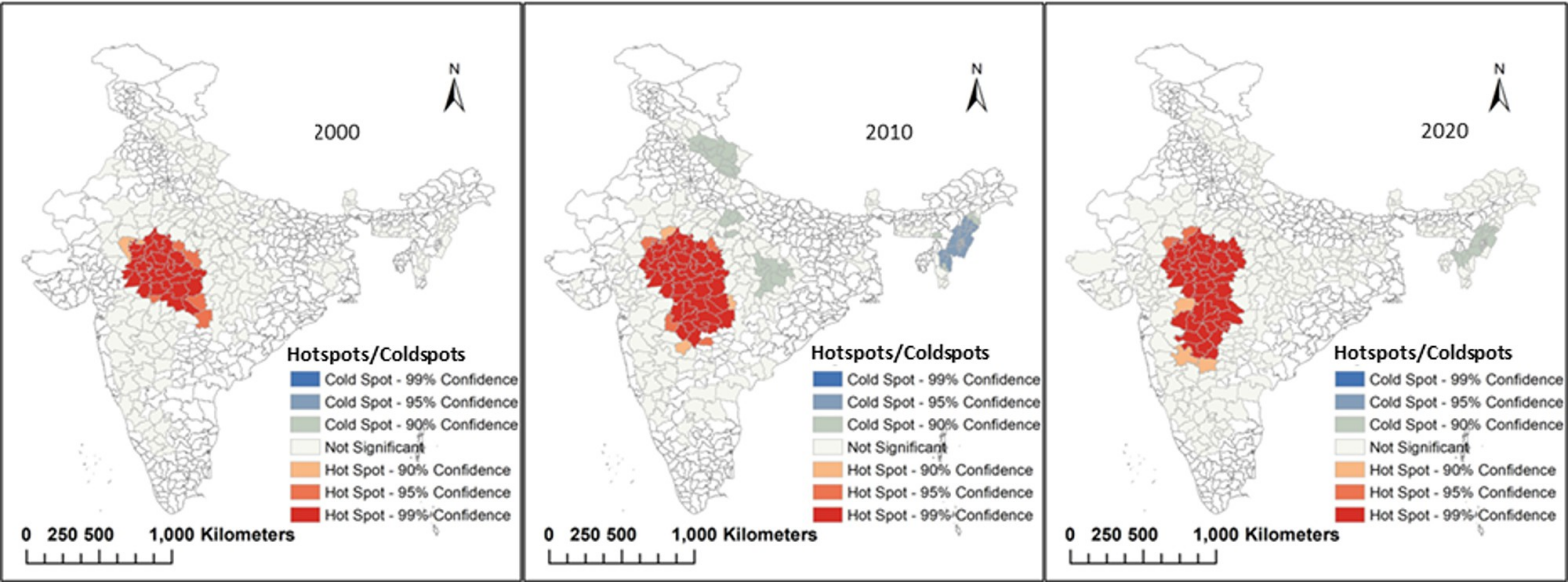
Hotspots and coldspots of soybean production in c. 2010, c. 2000 and c. 2020.

The hotspot maps generated using production statistics (Fig 6) shows that in c. 2000 the soybean was concentrated in the Malwa region of Madhya Pradesh and it was major contributor to national production but in c. 2020 the soybean hotspot had spread to the western plateau hill region and covered some of the districts of Maharashtra and Rajasthan. The Malwa region of central India is no longer the major hotspot of soybean production as it was in the 1970’s, as the entire central belt of Madhya Pradesh and Maharashtra combined can now be called ‘the soybean bowl’. There has also been a trend in soy expansion towards western India, which includes the eastern districts of Gujarat and Rajasthan. The cold spots where low values cluster (99% confidence) are observed in north-eastern districts which means this region has the lowest producing districts as compared to the major producers. As of 2020, 42 districts are hotspots of soybean production, up from 29 in 2000.

**Fig 7:**
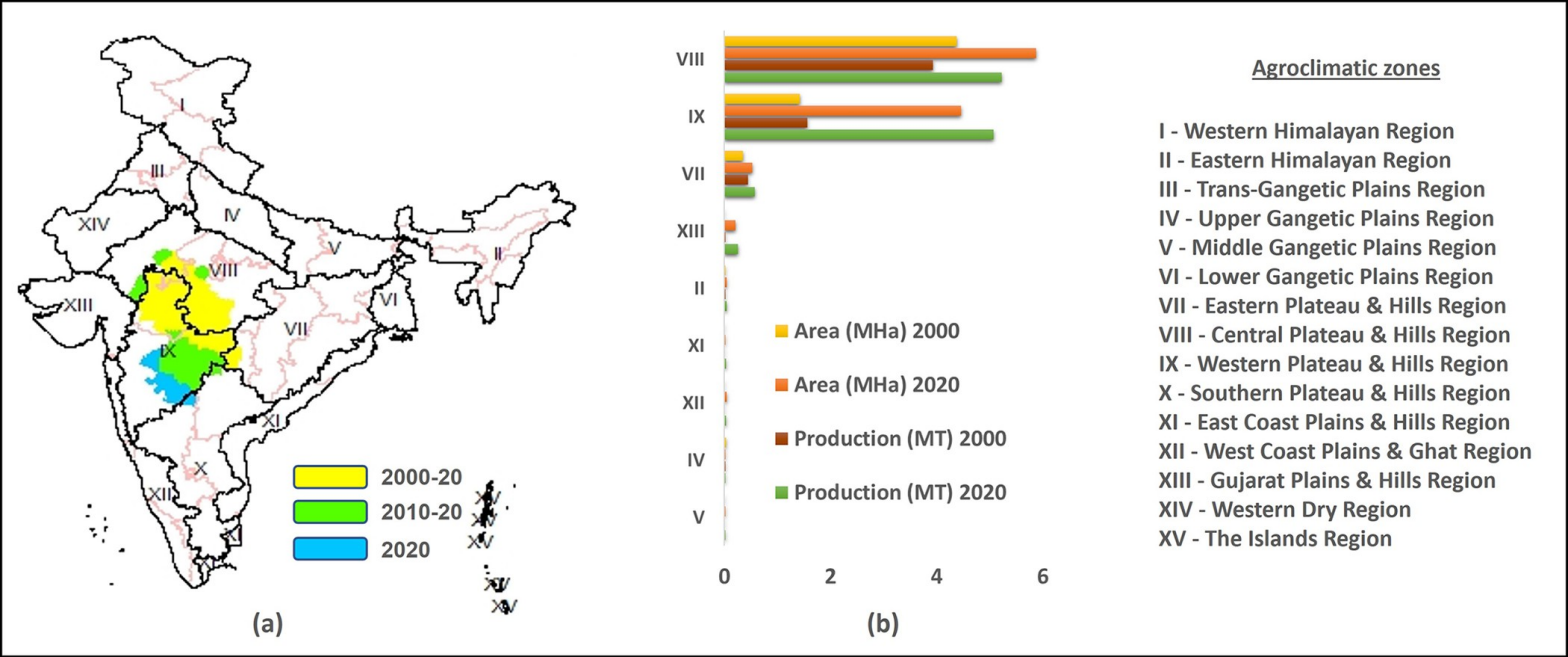
Hotspot expansion in three decades and distribution of soybean in agro-climatic zones. (a) Hotspot gradient diagram depicting the hotspot expansion over three decades; Yellow represents the soybean production hotspot areas for 2 consecutive decades (2000 to 2020); green and yellow together represents the soybean production hotspot districts for the last decade (2010 to 2020), and blue represents the districts that recently became hotspots i.e. c.2020. The red polygon represents the Indian state boundary while the polygons in black color represents the agroclimatic zones. The hotspots identified with 99% confidence level using Getis Ord G* statistics in ArcGIS. (b) The bar graph representing the area and production of soybean for various agroclimatic zones of India for c2000 and c.2020. The zones not producing soybean at all are not included in this graph.

Fig 7 shows that the hotspot districts are mainly concentrated in two agroclimatic zones on India viz. Central Plateau and Hills Region and Western Plateau and Hills as shown in the Fig 7(A). Agroclimatic zones can help identify areas that may be susceptible to specific weather-related hazards such as droughts, floods or frost, allowing farmers to take steps to mitigate those risks. India is divided broadly into fifteen agroclimatic zones based on soil, water, temperature conditions and agricultural practices. Soybean is mainly grown in three agroclimatic zones of India viz.

- Central Plateau and Hills Region (Aravali Malwa upland) (50 cm-100 cm)
- Western Plateau and hills (Maharashtra plateau region) (Rainfall 25-75cm)
- Eastern plateau and Hills Region (Southeastern Plateau region) (Rainfall 80-150cm)

Overall average annual rainfall from 25 cm-150 cm and soil types in these three zones are mixed red, yellow and black. From Fig 7(B) it can be seen that in the two decades, the crop has expanded to the Gujarat Plains and Hills, which has rainfall ranging from 50-100cm. In the Western Plateau and Hills, soybean production has tripled from 1.6 MT to 5.1 MT over the past two decades, while it has increased only by 35% in Central Plateau and Hills region from 3.9 MT to 5.2 MT. We overlaid the hotpots for 3 circa years with the agroclimatic zones (Fig 7) and found that hotspots of soybean are concentrated in two agroclimatic zones only i.e. Central Plateau and Hills Region (Aravali Malwa upland) and Western Plateau and hills (Maharashtra plateau region). For c. 2020 and the average yield of Western Plateau and Hills is 1.1 T/Ha and for the Central Plateau and Hills region is 0.8 T/Ha for c.2020, indicating that the former provided more favorable conditions for soybean cultivation during recent years.

The spatial mean of the production was calculated using the mean distance and showed that the spatial mean of soybean production has moved towards south by approximately 91 km and west by 24 km. In 2000, the center of overall hotspot of soybean was the Dewas District, while at present in c. 2020, the mean has moved 93 km to the south to Khandwa (East Nimar) District of Madhya Pradesh, which started the cultivation of soybean two decades ago.

### Drivers of trends and future projections

To better understand future prospects, in this section we address the drivers of soybean consumption in India and its future projections. We used long term population, price, import, export, domestic production and GDP per capita to examine the relation with the soybean consumption in India. Soybean is mainly consumed as edible oil and soy meal in India and hence we have performed regression and correlation analysis separately for soybean oil and meal. Domestic soybean oil and meal dynamics are influenced by multiple factors, including dietary habits and preferences, economic factors, and government policies like subsidies. Unfortunately, these data are not currently available at the national scale and were therefore not considered in this study. However, in future research, incorporating such ancillary variables could provide valuable insights into the drivers and future projections of oil and meal consumption. To achieve this, conducting a comprehensive national survey with robust sampling techniques will be essential for obtaining the necessary data. For this study we performed regression and correlation analysis with all the available relevant variables at national scale. Table 2(A) and 2(B) explains the correlation of soy oil and soy meal consumption with GDP per capita, import, export, production, population and commodity price while Table 2(C) and 2(D) shows the regression results of these five variables for soybean oil and meal consumption respectively. (The summary of regression results can be found in S5 to S7 Tables in S2 File).

Soy oil domestic consumption is highly positively correlated with population (0.904) and GDP per capita (0.962), meaning that as population and GDP per capita increase, the domestic consumption of soy oil also tends to increase. Soy oil price has a moderate positive correlation with soy oil Domestic consumption (0.600).

**Table 2:** Potential drivers of soybean expansion in India. (a) Pearson correlation coefficient matrix for soybean oil consumption, population, GDP per capita, import, export, and soybean oil price. (b) Results of a multiple linear regression analysis with soy oil as the dependent variable and population, GDP per capita, import, export, and soy oil price as independent variables. The table provides the coefficients, standard errors, t-statistics, and p-values for each independent variable, as well as the intercept. (c) Pearson correlation coefficient matrix for soybean meal consumption, population, GDP per capita, import, export, and soybean meal price. (d) Results of a multiple linear regression analysis with soybean meal as the dependent variable and population, GDP per capita, import, export and soy meal price as independent variables. The table provides the coefficients, standard errors, t-statistics, and p-values for each independent variable, as well as the intercept.

(a)									
* *	*Oil Domestic consumption*	*Oil Production*	*Oil Import*	*Oil Export*		*Oil price*	*Population*		*GDP Per capita*
Oil Domestic consumption	1								
Oil Production	0.861	1							
Oil Import	0.960	0.687	1						
Oil Export	0.594	0.566	0.551	1					
Oil price	0.600	0.737	0.459	0.270		1			
Population	0.904	0.954	0.771	0.587		0.636	1		
GDP Per capita	0.962	0.883	0.890	0.549		0.706	0.886		1
(b)									
* *	*Coefficients*		*Standard Error*			*t Stat*			*P-value*
Intercept	-161.959		100.225			-1.616			0.113
Oil Production	0.824		0.062			13.398			2.593E-17
Oil Import	0.931		0.022			42.101			8.658E-38
Oil Export	-3.335		2.057			-1.621			0.112
Oil price	-233.754		134.520			-1.738			0.089
Population	2.81E-07		1.32E-07			2.124			0.039
GDP Per capita	0.220		0.058			3.804			4.26E-04
(c)									
* *	*Meal Domestic consumption*	*Soy Meal Production*	*Meal Import*	*Meal Export*		*Meal price*	*Population*		*GDP Per capita*
Meal Domestic consumption	1								
Meal Production	0.836	1							
Meal Import	0.559	0.373	1						
Meal Export	0.341	0.797	0.053	1					
Meal price	0.728	0.758	0.380	0.493		1			
Population	0.859	0.954	0.372	0.698		0.689	1		
GDP Per capita	0.973	0.883	0.519	0.453		0.780	0.886		1
(d)									
* *	*Coefficients*		*Standard Error*		*t Stat*			*P-value*	
Intercept	-312.996		224.384		-1.395			0.170	
Soy Meal Production	0.749		0.053		14.063			4.42E-18	
Meal Import	0.908		0.313		2.901			0.006	
Meal Export	-0.806		0.043		-18.741			6.94E-23	
Soy Meal price	-16.887		12.417		-1.360			0.181	
Population	4.61E-07		2.81E-07		1.638			0.108	
GDP Per capita	0.658		0.150		4.394			6.71E-05	

The correlation analysis (Table 2(A)) reveals significant positive relationships between soybean oil consumption and domestic oil production, oil import, population, and GDP per capita. However, no significant correlation is found between soybean oil consumption and oil export or soy oil price, likely due to the influence of external factors such as market dynamics and international trade policies. Additionally, the dataset’s limited scope may not capture the full complexity of these interactions. Hence, to gain a deeper understanding, we conducted regression analysis (Table 2(B), and the results indicate that soy oil production, oil import, population, and GDP per capita are significant predictors of soybean oil consumption. A high F-statistic with a very low p-value indicates that the model is statistically significant and the independent variables together are strong predictors of soybean oil consumption. While it is intuitive that demand would lead to more import and domestic production of soybean oil, it’s interesting to notice that population and GDP per capita significantly influence oil consumption, indicating improved affordability for soybean oil among a larger population may lead to more consumption and hence demand.

There is no projection available for GDP per capita, price, import and export of soybean oil, but according to the World Bank, the projected population of India in 2050 is approximately 1.67 billion people. Hence, we performed the regression for soybean oil domestic consumption with population only and results are shown in Table 3.

**Table 3:** Regression matrix showing relation between soy oil consumption and population.

	Coefficients	Standard Error	t Stat	P-value
Intercept	-3867.44	385.8129	-10.0241	1.48E-13
Population	5.74E-06	3.83E-07	14.98831	3.95E-20

The regression statistics show that there is a strong positive correlation between domestic soybean oil consumption and population. The coefficient of determination (R-squared) is 0.8179, indicating that 81.8% of the variation in soybean oil consumption can be explained by the variation in population. The adjusted R-squared value of 0.8143 suggests that the model is a good fit for the data. The regression equation for this model is:

DomesticSoybeanOilConsumption=‐3867.44+5.74E‐06xPopulation

Based on the above regression equation and assuming a projected population of 1.67 billion, the estimated soybean oil consumption in India in 2050 would be around 5.72 MT compared to the current consumption which is around 5 MT. It should be noted that this is just an estimate and there are many other factors that could impact soybean oil consumption in the future. At present, India imports around 3MT of soybean oil worth approximately 5 billion U.S dollars.

Correlation and regression analysis for soy meal consumption in India (Table 2(C) and 2(D)), suggests strong positive relationships with meal production, population, and GDP per capita, indicating that these factors play significant roles in driving domestic consumption. Additionally, there is a moderate positive correlation with meal import and meal price, suggesting their potential influence. However, the correlation with meal export is relatively weak, implying limited impact on domestic consumption. Regression analysis for soy meal consumption shows that soy meal production and imports exhibit a strong association with soy meal consumption, which is intuitive as heightened demand corresponds to increased domestic consumption and imports, ensuring a sufficient supply to meet growing consumer needs. The regression coefficient for GDP Per capita is positive (0.658) and highly statistically significant (p-value: 6.71E-05), indicating that as GDP per capita increases, soybean meal consumption also increases, implying greater purchasing power and affordability, leading to increased consumption of soybean meal as a protein-rich food source. While it would be valuable to forecast soybean meal consumption in India, our limitations lie in the absence of future projections for variables other than population. Furthermore, the statistical significance of population as a predictor is not sufficiently strong to base estimates solely on population data. However, conducting future analyses with more comprehensive data, including subnational level long-term consumption data, dietary preferences, population trends, and subnational trade data, could lead to more reliable predictions of soybean meal as well as soybean oil consumption in India.

### Indian soybean production in an international context

**Fig 8:**
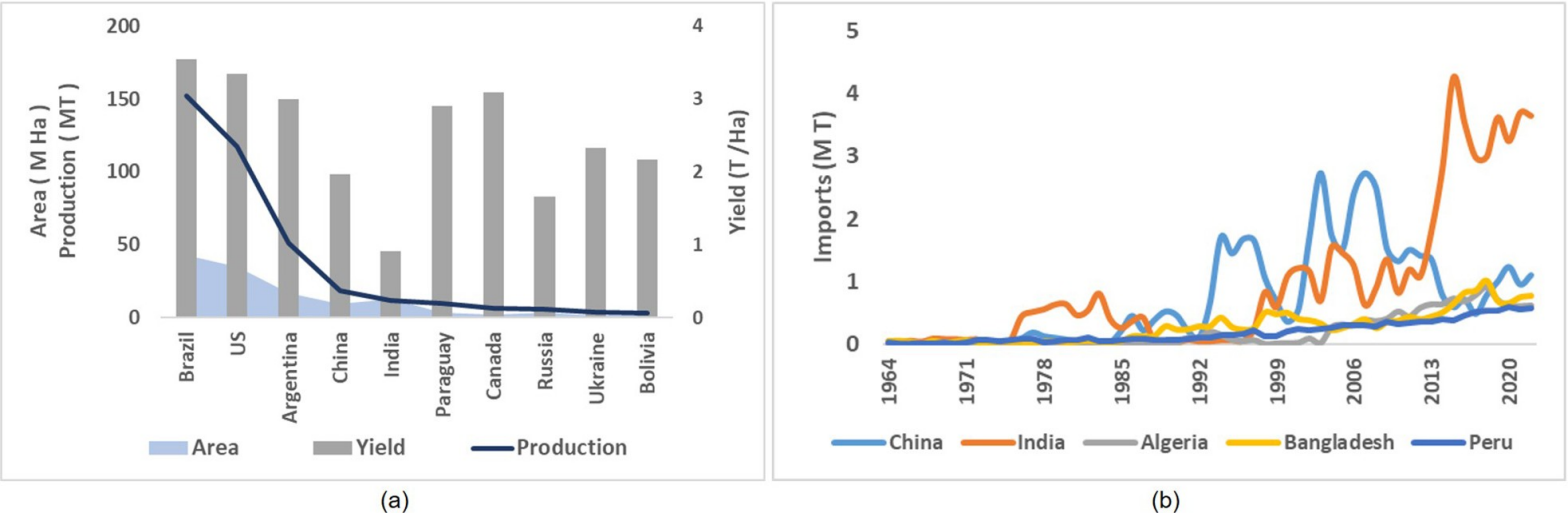
Comparison with international top producers and consumers of soybean. (a) Area, production and yield for major 10 soybean producers of the world. (b) Major soybean oil importing countries of the world.

Placing Indian soybean production and demand in an international context is crucial. It can help identify global trends in soybean production and consumption, which can aid in predicting future demand and supply dynamics. Soybean contributes around 30% of the worlds current edible oil need. Major producers of soybean oil are China, USA, Brazil and Argentina, with India ranked 5th in production (Fig 8(A)). Major importers of soybean oil include China, India, Algeria, Bangladesh and Peru (Fig 8(B)). India is the world’s largest importer of soybean oil, surpassing China in 2013–14 (Fig 8(B)) despite being one of the major edible oil producers. This can mainly be attributed to the low production of soybean vis-à-vis the demand in India to feed its population (Fig 8(A)). The contribution of India in terms of the world’s soybean area is 10%, but the contribution to total world soybean grain is only 4%, indicating lower levels of productivity of the crop in India (<1 T/ha), as compared to other countries (world average 2 T/ha). Hypothetically, if the average soy yield for India was greater than 3 T/ha, and keeping other countries’ area and yield constant, India would become the 3rd largest producer, surpassing Argentina and China. Furthermore, the productivity of soybean crop in India is low, as compared to the other countries e.g., Brazil, USA, China and Argentina (Fig 8(A)) majorly because of its smaller duration to maturity (90–120 days) as compared to Brazil, Argentina and USA (140–160 days) and relatively unfavorable climatic and soil conditions. India needs to invest heavily in research and development of soybean varieties that are well-suited to its local growing conditions to be able to meet its domestic demands without increasingly depending on imports.

## Discussion

India is the 4th largest country in terms of soybean acreage but it is 5th largest soybean producer in the world. This is due to its relatively poor productivity, as compared to the major soy producers which is a matter of concern for the policy makers to include and implement corrective strategies under the “Self-reliant-India” (Atma-Nirbhar Bharat) program, presently in operation throughout the country. The average productivity is less than 1T/Ha while the potential yield as proven by some studies, is more than 3 T/Ha [24]. Hypothetically, if the average productivity is more than or equal to 3 T/Ha, India could become the third largest producer in the world if other countries yield and area remain constant. Improving productivity is important for India, particularly as since 2013 it has by far been the world’s largest edible oil importer. Hence, it is important to understand the potential for oilseed expansion for the country to become self-sufficient in oilseed production. Since its introduction in the 1970’s, soybean has proven to be one of the most successful oilseeds in the country. Knowing the spatial dynamics and trends in soybean production is important when considering potential enhancements, and targeted interventions eventually helping farmers and the country’s economy in the context of national food security.

We have shown that overall yield improved within a few years from when it was introduced in 1970 (0.5 T/Ha), however after these initial improvements, national average yield has remained around 1 T/Ha. At the state level, Maharashtra and Gujarat have shown slightly better yields of 1.2 T/ha, while Madhya Pradesh and Rajasthan remain below 1 T/ha. This study shows that soy yield is better in the northeastern districts of India followed by Deccan Interior and Maharashtra Plateau region, while the previously major producer state of Madhya Pradesh has many districts that are now reporting the poorest yield. It appears that the prevailing climate (especially the monsoon distribution vis-à-vis seasonal drought) has had more of an impact in central India, as compared to other regions with districts with high yields or that the varieties or practices being used need to be improved [25, 26]. In this context, it is worth noting that the majority of the farmers in central India prefer growing short duration soybean varieties like JS 95–60, which is the dominant cultivar grown in more than 90% area and is found to be adversely affected by insect-pests and diseases, causing heavy yield losses in addition to the vagaries of monsoon [27]. Therefore, a countrywide survey is needed to find out the underlying reasons for the low productivity and region-specific plans will be needed in order to improve the overall soybean output. The potential yield of India’s soybean is reported to be more than 3 T/Ha, as has been observed from the frontline demonstrations conducted across the country, whereas the actual yield averages out to less than 1T / Ha [16]. This significant yield gap can be attributed to various factors including but not limited to farming practices, poor adaptation of technology, timely availability and affordability of quality inputs, biotic and abiotic stresses as reported by recent studies [10, 28].

Looking to the success of soybean in the central India along with its contribution to the socio-economic transformation of farmers, its cultivation has been adopted by farmers of the neighboring states of Maharashtra, Rajasthan and Karnataka, a trend of increasing trends expansion. From 1970 to 2000 Madhya Pradesh contributed more than 97% of total soybean of the country but in recent decades, its relative contribution has halved to 44%, while production in Maharashtra now contributes 44%, hence Madhya Pradesh is no longer the only major producer. The decadal growth rate (CAGR) was positive for the initial four decades, but turned negative for the recent decade, likely due to poor yield likely associated with adverse weather conditions. When we compared the area, production and yield by agroclimatic zone, we found that soybean is mainly concentrated in three agroclimatic zones, dominated by the Aravali Malwa upland region. District-level analysis showed that the total number of districts constituting hotspots of soybean cultivation in India have increased from 29 to 42 in three decades. Ujjain District (Madhya Pradesh) the highest producer for three decades, has been recently overtaken by the Buldana District (Maharashtra) and production in Maharashtra has been fast-growing, as compared to Madhya Pradesh. In Maharashtra, soy was adopted as intercrop and later as pure crop with replacement of less remunerative crops, that also slowed down the growth observed in first two decades. In Maharashtra, the intercropping of soybean with pigeon-pea is most common practice followed since many years by the farmers of Vidarbha and Marathwada region. Being most remunerative cropping pattern, it has replaced less remunerative crops such as sorghum, bajra, cotton, rice [29–31]. While the state of Madhya Pradesh has faced more adverse effects from climate change in recent years, lower soybean yields in the state can also be attributed to poor crop management, especially in terms of nutrient management of this energy packed crop in terms of protein and oil. Additionally, the farmers in Maharashtra appear to be more receptive to adopting agricultural technology compared to their counterparts in Madhya Pradesh. According to a recent study, the mean score of practices adopted by farmers in Maharashtra is 12.4, whereas it is only 11 for farmers in Madhya Pradesh [32]. The other states with soybean production are Karnataka, Telangana, Rajasthan and Gujarat, together contributing to 12% of total production. The hotspot analysis shows that the hotspot of soybean production is concentrated in two agroclimatic zones only i.e., Central Plateau and Hills Region (Aravali Malwa upland) and Western Plateau and hills and center of crop is moving towards the west and south rapidly.

We examined the drivers of soybean consumption in India, specifically analyzing the correlation and regression between population, GDP per capita, import, export, and commodity prices with soybean oil and meal consumption. Results show a strong positive correlation between domestic soy oil consumption and population and GDP per capita. Regression analysis indicates that 95.1% of the variation in domestic oil consumption can be explained by the three predictor variables. Based on assumptions and projections, the article estimates an increase in domestic soy oil consumption to approximately 5.7 MT by 2050 due to population growth. India currently imports around 3 MT of soybean oil at present worth US $5 billion approximately. Therefore, it is imperative to enhance soybean production either by expanding the area under cultivation or improving productivity to reduce dependence on imports. Understanding the temporal and spatial distribution of soybean production in relation to other crops, provides an important step towards developing national and state policies and actions to increase national food security. Developing and targeting sustainable solutions to reduce the yield gaps identified in this study will help both farmers and the national economy. The likelihood of extreme weather and the impacts of climate change mean that such solutions will need to be climate resilient. Solutions will also need to be holistic in terms of cropping systems and competing crops, with a view to meeting nutritional needs, ensuring farm level economic resilience and national supply and demand. Towards this, the ICAR-Indian Institute of Soybean Research, Indore in association with more than 32 centers operating in different soybean growing states has intensified its varietal development programs. It has recently developed more than 35 soybean varieties during the last three years, suiting different agro-ecological conditions. These include varieties having varying maturity duration, resistance to biotic factors like insect pests and diseases, and also waterlogging & drought tolerance. However, adaptation of these varieties by farmers on a large scale would take time because the seed production chain can only commence after receiving the gazette notification, which authorizes their commercial cultivation and distribution, ensuring they meet all the necessary standards [33].

Consistent and dependable agricultural statistics are crucial for conducting a comprehensive analysis. However, the time gap between the statistical survey and data availability necessitates the use of other methods to offer the government timely information on soybean production for export and import decisions. To this end, satellite remote sensing may offer a solution to crop monitoring in near real-time. Nevertheless, persistent cloud cover during monsoon (kharif) season creates a challenge, and thus, it is necessary to develop novel microwave satellite data-based methods for monitoring monsoon crops including soybean [34].

## Conclusion

Soybean cultivation in India has a relatively short commercial history, with its introduction in central India during the 1970s. However, it has since become a significant agricultural commodity and an important source of oilseeds. Given India’s position as the world’s largest importer of edible oil and the challenges of national food security and a fast-growing population, national soybean production is a matter of great importance. With increasing affordability, a growing health consciousness, and a rising population, the demand for soybeans is expected to rise in the coming years. Soybeans have the potential to reduce malnutrition and improve food security in India due to their protein content. A novel spatial statistical analysis of soybean distributions in India is presented in this study using national, state, and district level spatial statistical models. The analysis shows an increase in soybean production across the country, but the annual growth rate has declined over consecutive decades, even as the cultivation area expands. We found that soybean cultivation has expanded significantly towards southern and western India. The hotspot regions of high soybean production remain the Malwa Upland and Maharashtra Plateau region, while cold spots (low production) are found in the north eastern regions of India, with considerable overall variability. Initially Madhya Pradesh was a major contributor of soybean in India which is now being matched by Maharashtra and other neighboring states also rapidly adopting the crop. The once major producer districts are no longer yielding good soybean output, while new districts are delivering a better yield. While some districts report high soybean yields (more than 3 T/ha), the national average yield is around 1 T/ha, and many major producing districts have yields below 1.5 T/ha. The soybean yield in India is quite low and unstable throughout the country hence making it important to conduct comprehensive studies to investigate the causes of the low and unstable soybean yield in India. Recent years have shown drastic decreases in yield in major producing districts due to unusually erratic rains and droughts in successive years, hence it is needed to take measures to help develop varieties that are both higher yielding and climate resilient. Improved soybean production in India can improve the economy and food security and would involve closing the yield gap and region-specific plans to improve the low productivity of soybean cultivation. By addressing data challenges and adopting sophisticated analytical approaches in this study, we anticipate contributing significantly to the understanding of soybean cultivation and its implications for India’s edible oil self-sufficiency and global competitiveness.

## Supporting information

S1 File Inclusivity in global research. (DOCX)

S2 File Supplementary Tables (S1-S7 Tables). (XLSX)
